# Effects of the COVID-19 Pandemic on Access to Education and Social Participation in Children and Adolescents with Duchenne Muscular Dystrophy in Switzerland

**DOI:** 10.1055/s-0043-1764434

**Published:** 2023-03-30

**Authors:** Bettina C. Henzi, Dominique Baumann, Sarah J. Erni, Nadine Lötscher, Anne Tscherter, Andrea Klein, on behalf of the Swiss-Reg-NMD Group

**Affiliations:** 1 Department of Pediatric Neurology and Developmental Medicine, University Children's Hospital Basel, University of Basel, Basel, Switzerland; 2 Institute of Social and Preventive Medicine, University of Bern, Bern, Switzerland; 3 Division of Neuropediatrics, Development and Rehabilitation, Department of Pediatrics, University Hospital of Bern (Inselspital), University of Bern, Bern, Switzerland

**Keywords:** Duchenne muscular dystrophy, COVID-19 pandemic, education, participation

## Abstract

Two-thirds of patients with Duchenne muscular dystrophy (DMD) have cognitive and neuropsychiatric problems. Concerning their quality of life, negative factors are the lack of qualifying education and social participation in sporting and leisure activities. Adapted assistance in education and participation in social life are thus important. During the coronavirus disease 2019 (COVID-19) pandemic, the pediatric population was less severely impacted by the disease, but by the restrictions associated. The aim of this study was to evaluate the impact of the COVID-19 pandemic regarding access to education and social participation for young patients with DMD in Switzerland. We conducted a survey study from May to August 2021 assessing the impact of the COVID-19 pandemic on access to education and social participation in 8 to 18 years old patients with DMD in Switzerland. Of 60 sent surveys, 40 were returned and included. Mean age of participants was 13.5 years (±3.1 standard deviation); 23/40 of the participants were wheelchair bound, 21/40 attended a special school, and 19/40 a regular school. Of the 22/40 participants receiving assistance at school, 7/40 reported a change caused by the pandemic: for 5/7, the assistance was paused. Of the 12 boys and adolescents attending sporting activities, 10 had to suspend these. Nine attended other leisure activities; for 3/9, these activities were paused. The COVID-19 pandemic had direct effects on school assistance, sporting, and leisure activities in young patients with DMD in Switzerland. It is important to ensure that school assistance and leisure activities are rapidly resumed.

## Introduction

Duchenne muscular dystrophy (DMD) is an X-linked recessive disorder affecting ∼1 in 3,500 live male births. 1 Mutations in the *DMD* gene cause missing or abnormal dystrophin. Clinically, DMD is characterized by progressive muscle weakness and severe restrictions of motor function including mobility. Depending on the mutation location, central nervous system symptoms are a common feature of DMD. 2 Around two-thirds of DMD patients present with cognitive impairment, and only few patients achieve an academic degree or can complete a professional training. 3 4 5

Self-perceived quality of life in DMD patients has been found to be comparable to nonaffected peers. An important negative factor is the lack of qualifying education, opportunities for leisure activities, vacation, and social participation. 6 7 8 Therefore, assistance in education, physical, and psychological support to pursue academic goals and to facilitate participation in social life are immensely important.

Coronavirus disease 2019 (COVID-19) became a pandemic and worldwide public health emergency. 9 Concomitant diseases were assumed to impose a higher morbidity from COVID-19 in the pediatric population. But in retrospect, the pediatric population was less affected by the disease itself in the first waves but suffered from restrictions impacting their daily life. 10 We hypothesized that COVID-19-related restrictions have a negative impact on access to education and social participation in DMD patients. We conducted a survey to evaluate access to education and social participation in children and adolescents with DMD in Switzerland to assess how the COVID-19 pandemic impacted on these aspects.

## Methods

The study took place in the framework of the Swiss Registry for Neuromuscular Disorders (Swiss-Reg-NMD). It is a population-based registry collecting health-related data from patients with a neuromuscular disease in Switzerland. It was approved by the local ethics committee (June 20, 2018, KEK Bern, 2018-00289). After written informed consent for the Swiss-Reg-NMD was obtained, medical data were collected, and patients were invited to participate in questionnaire studies. Participation in questionnaire studies is voluntary.

The primary objective of this study was to assess the impact of the COVID-19 pandemic on access to education and social participation in young DMD patients.

We included all patients registered in the Swiss-Reg-NMD diagnosed with DMD, aged 8 to 18 years, living in the German- or French-speaking part of Switzerland.

For this study, our questionnaire was focused on mobility, assistance at school, sporting activity outside of regular school curriculum, leisure, and vacation activity before and after the first lockdown in March 2020 due to the COVID-19 pandemic. According to the age of the child, different questionnaires were sent to the families. An information letter addressed to the patients and their families was enclosed. Additionally, the treating child neurologist was informed. We sent out the survey documents in May 2021 to all patients and their families and a reminder in June 2021.

Pseudonymized data obtained by the returned questionnaires were entered into the Research Electronic Data Capture (REDCap) database hosted by the Institute of Social and Preventive Medicine at the University of Bern, Switzerland. Statistical analysis was performed with SPSS (IBM SPSS Statistics Version 25). Descriptive statistics were calculated (percentage, mean, and standard deviation [SD]).

## Results

### Study Population

Of a total of 60 dispatched surveys, 42 (70%) were returned. Thereof, 40 (67%) questionnaires were filled out and could be included in this analysis. In some cases, single questions were not answered. Patients' age ranged from 8.3 to 17.8 years; mean age was 13.5 years (±3.1 SD).

### Ambulation and Participation in Sports

Of the 40 patients included in this analysis, 23/40 (58%) were wheelchair bound, 9/40 (22%) used their wheelchair for longer distances, and 8/40 (20%) were ambulant; 15/40 (38%) attended school sports classes, additional 4/40 (10%) participants attended with assistance, and another 4/40 (10%) attended only partially; 16/40 participants (40%) did not attend school sports classes.

### Special Needs School and Assistance at School

A total of 21/40 (52%) attended a special needs school and 19/40 (48%) a regular school. Of the latter, seven received assistance for mobility purposes only and four for assistance in learning for various subjects and 1 for both. Of the 21 students attending a special needs school, 10 received additional assistance in various areas such as speech therapy, support for mobility, and special needs education.

**Fig. 1:**
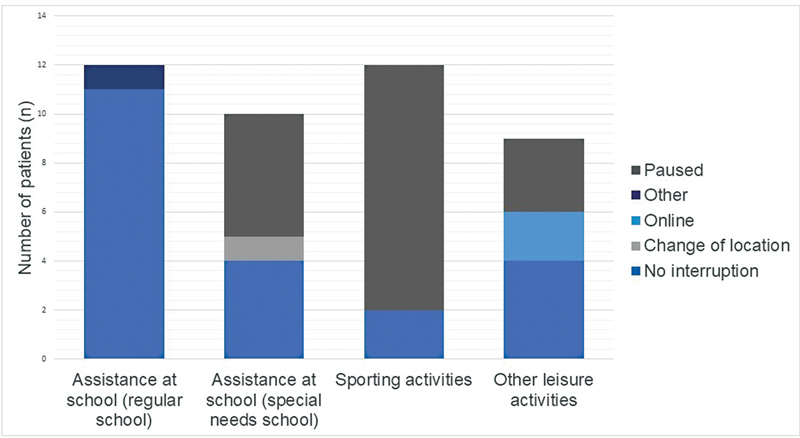
Effects of the coronavirus disease 2019 pandemic on access to education and social participation in young people with Duchenne muscular dystrophy in Switzerland from March 2020 to May 2021.

Of the 22 patients who received assistance at school, 7 (32%) reported a change caused by the COVID-19 pandemic ( Fig. 1 ). In 5/7, the assistance was paused or stopped, in one a change of location occurred and one student reported a change due to hygienic measures (e.g., necessity to wear a face mask). Five of the seven respondents who reported a change were attending a school for special needs.

### Sporting and Leisure Activities

Of the 40 participants, 12/40 (30%) participated in sporting activities outside the school curriculum before the lockdown in March 2020. Sporting activities were paused in 10 of 12 (83%) cases due to the COVID-19 pandemic; 9/40 (22%) participants attended leisure activities such as scouting, club membership, or music bands. Thereof, for 3/9 (33%) participants, the activity was paused due to the COVID-19 pandemic, and for 2/9 (22%) participants, the activities were shifted to virtual space ( Fig. 1 ).

Eight (20%) of the 40 participants spent their summer or fall vacation 2019 in a specialized camp. In spring 2020, 33/40 (83%) participants did not go on any vacation, and only 1 patient spent time in a specialized vacation camp. No summer camp took place in summer 2020, and only one patient attended a vacation camp again in spring 2021.

## Discussion

In the present survey study assessing the impact of the COVID-19 pandemic on access to education and social participation in young patients with DMD in Switzerland, we observed that of the 22 boys and adolescents requiring assistance at school, in 5 (23%) cases, this assistance has been paused due to the COVID-19 pandemic and its associated restrictions. The proportion of halted sporting activities is even higher, as 10 (83%) of 12 boys and adolescents had to pause their sporting activity all together.

In Switzerland, schools were closed due to the COVID-19 pandemic for 2 months in spring 2020. In May 2020, schools were reopened in a step-like arrangement. The Swiss Society of Pediatrics recommended in several public statements that schools were to be left open whenever possible. Different European countries handled school openings during the COVID-19 pandemic differently, but over the course of the pandemic, the concern about secondary educational and psychological consequences for children and adolescents increased. 11 12 Young patients with underlying diseases found themselves in a difficult situation especially in the beginning of the pandemic. On one hand, they were grouped as patients at an increased risk for a COVID-19 infection and its complications, and on the other hand, education and social participation are as important as for healthy peers. Assistance facilitating their participation in school or other activities is indispensable.

We see in our analysis that the COVID-19 pandemic had a substantial effect on our patients with DMD with reduced assistance at school. Remarkably, in our cohort, participants attending a special needs schools appear to be affected more than those attending a regular school. Moreover, sporting activities suffered an important shortening as well. We postulate that these interruptions have to be held to a minimum to prevent further drawback in this vulnerable group of young people.

A limitation of our study is the lack of a control group. The impact of the COVID-19 pandemic on other patient cohorts with neuropediatric diseases was studied for children with epilepsy. In these, delayed access to medical care led to poor seizure control. 13 The impact of delayed consultation in neuromuscular patients was not evident in our cohort.

In several studies, the effects of the COVID-19-associated restrictions on learning, 14 mental health, physical activity, and sedentary behavior in healthy children and adolescents were highlighted and appear to be substantial like in our cohort.

Furthermore, over the course of the COVID-19 pandemic, it appears that the effects of a COVID-19 infection in children with neuromuscular disorders may not be as severe as expected. 15 Until August 2021, two (5%) patients from our cohort were reported to have had COVID-19 infection with only mild symptoms. To our knowledge, there were no patients in our cohort with a severe COVID-19 infection needing admission to intensive care. In the beginning of the COVID-19 pandemic, the justification for reducing school attendance was to protect this potentially vulnerable cohort. In retrospective, we see secondary problems due to the restrictions associated with the COVID-19 pandemic becoming more important like in the healthy population. Based on our preliminary study, we assume that the benefits of good access to education and social participation may outweigh the risks of a COVID-19 infection, which has to be further analyzed in depth. With this in mind, we recommend to actively support patients and their families to re-establish their usual assistance in school and leisure activities.

**Conclusion** The COVID-19 pandemic had direct effects on school assistance, sporting, and leisure activities in young patients with DMD in Switzerland. It is important to ensure that school assistance and leisure activities are rapidly resumed.

## Notes

Appendix A Swiss-Reg-NMD group
Berenice Bubl, ^4^ Andrea Capone, ^5^ Cornelia Enzmann, ^1,5^ Joel Fluss, ^6^ Oswald Hasselmann, ^7^ David Jacquier, ^8^ Hans H. Jung, ^9^ Andrea Klein, ^1,3^ Petra Kolditz, ^10^ Claudia E. Kuehni, ^2,11^ Gian P. Ramelli, ^12^ Paolo Ripellino, ^13^ Oliver Scheidegger, ^14^ Bettina Schreiner, ^9^ Esther I. Schwarz, ^15^ Georg M. Stettner ^16^
Affiliations of the Swiss-Reg-NMD group:

^4^ Division of Neuropediatrics, Luzerner Kantonsspital, Luzern, Switzerland
^5^ Division of Pediatric Neurology, Children's Hospital, Aarau, Switzerland
^6^ Pediatric Neurology Unit, Geneva Children's Hospital, Geneva, Switzerland
^7^ Department of Neuropediatrics, Children's Hospital of Eastern Switzerland, St. Gallen, Switzerland
^8^ Pediatric Neurology and Neurorehabilitation Unit, Lausanne University Hospital, Lausanne, Switzerland
^9^ Department of Neurology, University Hospital Zurich, Zurich, Switzerland
^10^ Kinderarztpraxis Alpenquai, Lucerne, Switzerland
^11^ Children's University Hospital, Inselspital, University of Bern, Bern, Switzerland
^12^ Neuropediatric Unit, Pediatric Institute of Southern Switzerland, Ospedale San Giovanni, Bellinzona, Switzerland
^13^ Neurocenter of Southern Switzerland, Lugano, Switzerland
^14^ Department of Neurology, Centre for Neuromuscular Diseases, Inselspital, Bern University Hospital, University of Bern, Bern, Switzerland
^15^ Department of Respiratory Medicine, Sleep Disorders Centre and Neuromuscular Centre, University Hospital of Zurich, Zurich, Switzerland
^16^ Department of Pediatric Neurology, Neuromuscular Center, University Children's Hospital Zurich, University of Zurich, Zurich, Switzerland
